# Impact of paramagnetic rim lesions on disability and race in multiple sclerosis: mediation analysis

**DOI:** 10.1002/acn3.52203

**Published:** 2024-09-17

**Authors:** Nara M. Michaelson, Sandra H. Rúa, Ulrike W. Kaunzner, Melanie Marcille, Iliana Pliska‐Bloch, Kimberly Markowitz, Thanh D. Nguyen, Susan A. Gauthier

**Affiliations:** ^1^ Department of Neurology Weill Cornell Medicine New York New York USA; ^2^ Department of Mathematics and Statistics Cleveland State University Cleveland Ohio USA; ^3^ Department of Radiology Weill Cornell Medicine New York New York USA; ^4^ Feil Family Brain and Mind Institute, Weill Cornell Medicine New York New York USA

## Abstract

**Objective:**

Black American (BA) multiple sclerosis (MS) patients experience greater disability compared to White American (WA) patients. Here, we investigated the role of paramagnetic rim lesions (PRLs), a subset of chronic active lesions, on race‐related disability in MS.

**Methods:**

We conducted a retrospective observational study comparing BA and WA MS patients. PRLs were identified through Quantitative Susceptibility Mapping (QSM) MRI. A causal mediation analysis explored the impact of PRLs on the relationship between race and disability, as measured by the Expanded Disability Status Scale (EDSS).

**Results:**

The prevalence of PRLs in BA patients with MS was higher at 55% compared to WA patients at 39% (*p* = 0.022). A higher percentage of PRLs among all white matter lesions was observed with BA (8.01%) patients compared to WA (3.4%) patients (*p* = 0.003). In a regression analysis, controlling for significant patient‐level covariates and income‐level demographics, the percentage of PRLs was, on average, 4.61 points higher for BA patients than for WA patients (*p* = 0.003). In a separate regression analysis, accounting for covariates, BA patients exhibited significantly higher EDSS scores (*p* < 0.001). Further analysis demonstrated that the percentage of PRLs was a mediator in the association between BA patients and greater disability (*p* = 0.031). Higher proportion of PRLs in BA population accounted for 14% of the total effect of race on disability.

**Interpretation:**

BA patients exhibit greater disability, in part, due to their higher proportion of PRLs. This study underscores the substantial impact of chronic active lesions on disability outcomes in this specific minority MS patient population.

## Introduction

Multiple sclerosis (MS) is an immune‐mediated disease affecting the central nervous system (CNS), leading to acute inflammatory events that result in focal demyelination and axonal loss. The disease is also characterized by persistent inflammation within chronic lesions, known as “smoldering inflammation,” which may play an important role in ongoing disease pathogenesis.^1^ There is a critical need to understand the varied contributions of key pathological processes among individual patients,^2^ with the aim of improving disability in understudied populations such as in Black Americans (BAs) with MS. Contrary to initial assumptions, recent studies have revealed that the prevalence of MS may actually be higher in non‐White compared to White Americans (WAs).^3^ Another distinction appears to be the difference in disease course between BAs and WAs with MS, with BAs having a younger age of onset and faster disability progression.^4^ BAs also exhibit higher levels of inflammatory markers in the cerebrospinal fluid (CSF),^5^ greater retinal tissue loss,^4^ higher lesion volume burden,^4^ and a greater degree of brain atrophy.^4^ There are significant issues related to health disparities among the BA population, as highlighted in a recent review article,^6^ which describes limited access to resources, underrepresentation in clinical studies, and reduced socioeconomic status. These observations underscore the necessity for more effective approaches to addressing MS disparities, which may include prioritizing higher efficacy treatments and developing more comprehensive care strategies for the BA minority population. In doing so, we can aim to mitigate and minimize the factors contributing to differences in disease course.

Recent advances in knowledge regarding the role of chronic active lesions in MS may help bridge the gap in our understanding of these race‐related disability differences. Smoldering inflammation present in chronic active lesions (CALs) appears to be a key factor in progressive cognitive and ambulatory decline in MS.^7^ We and others have validated that paramagnetic rim lesions (PRLs), which are a subset of CALs, have dense rims of iron‐laden pro‐inflammatory immune cells and represent sites of ongoing demyelination and axonal loss.^8^, ^9^ These lesions can be identified by iron‐sensitive MRI sequences such as high‐pass filtered phase imaging^10^ or quantitative susceptibility mapping (QSM).^11^ Compared to phase imaging, which is qualitative and susceptible to blooming artifacts, QSM is quantitative and provides more reliable localization of the iron source,^12^ and appears better able to identify PRLs with increased tissue damage.^13^ Studies have also shown the presence of PRLs are associated with increased clinical disability,^14^ involving worse cognitive functioning, as well as negative radiographic findings, such as cortical thinning and thalamic atrophy.^15^, ^16^ Patients with two or more PRLs were also found to have a higher level of serum neurofilament light chain (sNfL),^17^ which is a marker of neuronal loss. There have also been associations with other fluid biomarkers such as chitinase 3‐like‐1, a marker of astrocyte damage and macrophage/microglial activation. In patients with a first demyelinating event, there is a demonstrated correlation with the number of PRLs and CSF concentrations of chitinase 3‐like 1.^18^

As development of PRLs is associated with higher levels of disability,^19^ we hypothesize that these lesions may be connected with the more aggressive clinical phenotype and inflammatory profile observed in BA patients with MS. The objective of this study is to analyze differences in BAs and WAs with MS with regards to the prevalence of PRLs on QSM MRI and to understand whether this subset of CALs affects race‐related differences in disability.

## Methods

### Patient cohort and study design

**Figure 1:**
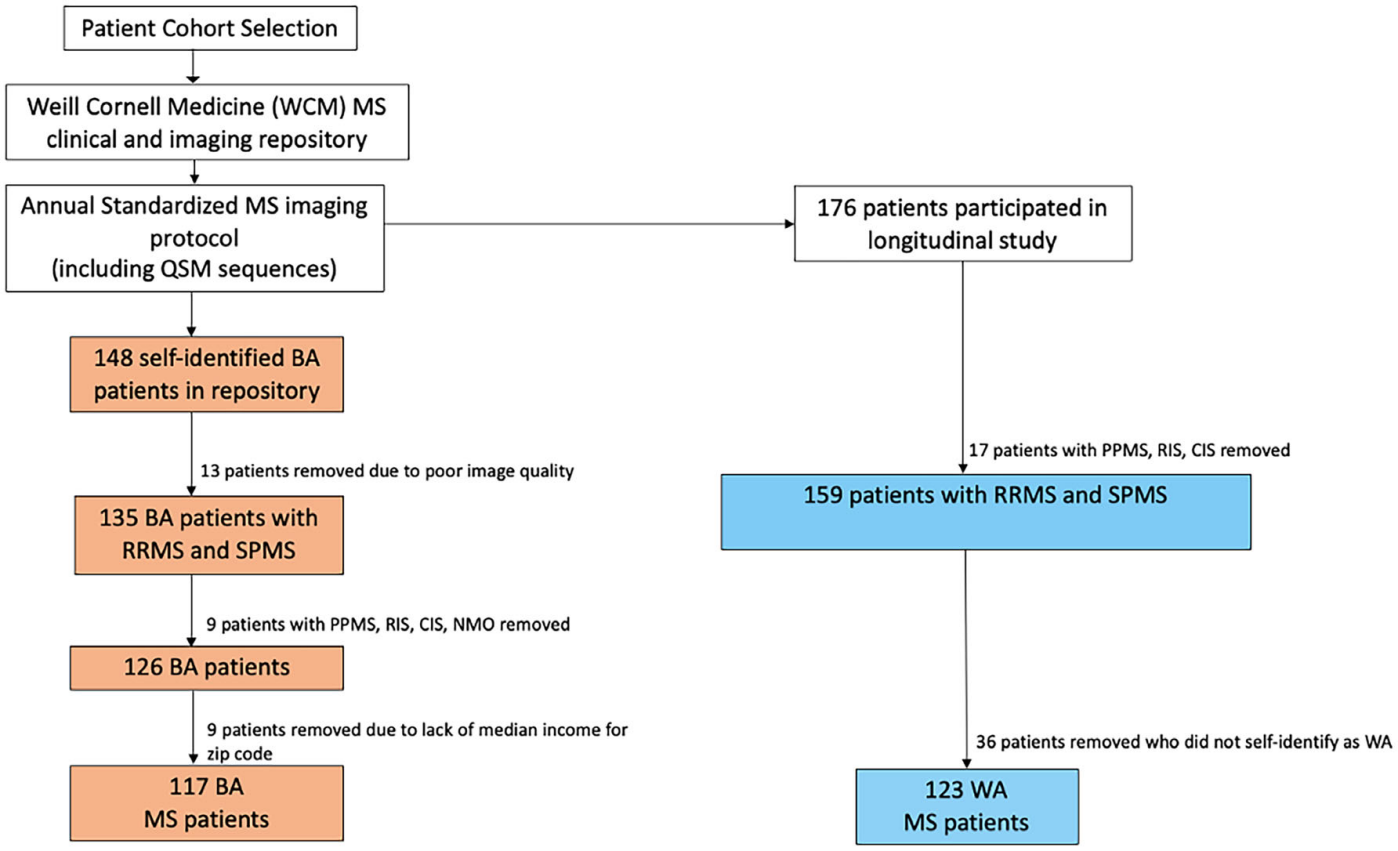
Study flowchart: Inclusion criteria in the cohort. Depicted are the BA and WA cohorts fulfilling the inclusion criteria. QSM, quantitative susceptibility mapping; BA, Black American; WA, White American; PPMS, primary progressive multiple sclerosis; RIS, radiologically isolated syndrome; CIS, clinically isolated syndrome; NMO, neuromyelitis optica.

This was a retrospective observational study designed to evaluate and compare the prevalence and impact of PRLs in a cohort of BA and WA patients with MS (Fig. 1). Patients with MS considered for this analysis met the following inclusion criteria: (1) the 2010 McDonald criteria,^20^ (2) diagnosed with either relapsing remitting (RR) or secondary progressive (SP) MS, (3) aged ≥18 years old, (4) already participants within our research repository, (5) underwent MRI imaging with QSM for PRL detection, and (6) self‐identified as BA or WA. Exclusion criteria were clinically isolated syndrome (CIS), radiologically isolated syndrome (RIS), neuromyelitis optica (NMO), and primary progressive multiple sclerosis (PPMS). The cohort of self‐identified WA MS patients was drawn from a registry previously used for published research at our institution (Fig. 1).^15^ Both WA and BA MS cohorts underwent MRI brain imaging with the majority of participants undergoing imaging under the same protocols and scanners (see section below on MRI protocol and Image Processing). There were no statistically significant differences between the demographics, clinical characteristics and treatment regimens of the two cohorts, with the exception of higher EDSS scores in BAs (Table 1). Additional clinical data included disease‐modifying therapy (DMT) type. DMTs were divided into lower and higher efficacy groups. Lower efficacy DMTs included interferon beta‐1a, interferon beta‐1b, glatiramer acetate, peginterferon beta‐1a, dimethyl fumarate, and teriflunomide. Higher efficacy DMTs included alemtuzumab, rituximab, ocrelizumab, fingolimod, and natalizumab. The Expanded Disability Status Scale (EDSS) score was used to measure neurologic disability. A higher EDSS score denoted higher disability level. Variables serving as proxies for socioeconomic status included zip code and associated income levels from census block‐level data for BA and WA demographics.

**Table 1:** Patient clinical, socioeconomic, and MRI characteristics of BA and WA patients with MS.

Characteristics	Black Americans (BA) with MS (*N* = 117)	White Americans (WA) with MS (*N* = 123)	*P*‐value
Male, *n* (%)	28 (23.9)	38 (30.9)	0.288
Disease duration (years), mean (SD)	11.00 (7.52)	10.60 (8.30)	0.550
Age (years), mean (SD)	44.55 (12.09)	43.00 (10.58)	0.238
Symptom onset to diagnosis (years), mean (SD)	2.65 (5.017)	2.33 (4.37)	0.609
Symptom onset to treatment start (years), mean (SD)	3.74 (5.705)	3 (5.22)	0.288
Median zip code income ($) mean (SD)	66085.81 (29486.83)	120000.00 (40388.69)	**<0.001**
MS subtypes			
RRMS, *n* (%)	115 (98.29%)	119 (96.65%)	0.725
SPMS, *n* (%)	2 (1.71%)	4 (3.25%)	
DMT type			
Lower/Medium efficacy	38 (32.44%)	41 (33.33%)	0.873
Higher efficacy	59 (50.43%)	64 (52.03%)	
Untreated	20 (17.13%)	18 (14.64%)	
EDSS, median (IQR)	2.00 [0.00, 3.00]	1.00 [0.00, 2.00]	**<0.001**
MRI measures			
Total FLAIR lesion volume, mean mm^3^, (SD)	9010.03 (10291.92)	4400.00 (6363.05)	**<0.001**
FLAIR lesion number, mean (SD)	23.83 (20.77)	22.00 (20.31)	0.487
PRL number, mean (SD)	1.32 (2.79)	0.86 (1.74)	0.130
Percentage of PRL lesions, mean (SD)	8.01 (15.25)	3.40 (7.50)	**0.003**
Total non‐PRL FLAIR lesion volume mean mm^3^, (SD)	8029.30 (9336.09)	3600.00 (5778.24)	**< 0.001**

BA, Black American; WA, White American; SD, standard deviation; MS, multiple sclerosis; RRMS, relapsing–remitting multiple sclerosis; DMT, disease‐modifying therapy; EDSS, expanded disability status scale; MRI, magnetic resonance imaging; FLAIR, fluid‐attenuated inversion recovery; PRL, paramagnetic rim lesion. Bold values have statistically significant *p* values.

This study was reviewed and approved by Weill Cornell Medicine Institutional Review Board, which is an ethics standards committee on human experimentation. Patients provided informed consent for the use of their data.

### MRI protocol and image processing

Imaging was performed on 3T Magnetom Skyra scanners (Siemens Medical Solutions USA, Malvern, PA, USA) using a product 20‐channel head/neck coil for all WA and the majority of BA (111/133 patients). MRI protocol consisted of sagittal 3D T1‐weighted (T1w), 3D T2w fluid‐attenuated inversion recovery (FLAIR), 2D T2‐weighted (T2w) fast spin echo sequence, gadolinium‐enhanced 3D T1w sequence, and axial 3D multi‐echo Gradient Recalled Echo (GRE) sequence for QSM. We used the same methodology as the one employed in a previous study,^15^ which includes the following specifications: (1) 3D sagittal T1w MPRAGE: repetition time (TR)/echo time (TE)/inversion time (TI) = 2300/2.3/900 ms, flip angle (FA) = 8°, GRAPPA parallel imaging factor (R) = 2, voxel size = 1.0 × 1.0 × 1.0 mm^3^; (2) 2D axial T2‐weighted (T2w) turbo spin echo: TR/TE = 5840/93 ms, FA = 90°, turbo factor = 18, R = 2, number of signal averages (NSA) = 2, voxel size = 0.5 × 0.5 × 3 mm^3^; (3) 3D sagittal fat‐saturated T2w fluid‐attenuated inversion recovery (FLAIR) SPACE: TR/TE/TI = 8500/391/2500 ms, FA = 90°, turbo factor = 278, R = 4, voxel size = 1.0 × 1.0 × 1.0 mm^3^; (4) axial 3D multi‐echo GRE sequence for QSM: axial field of view (FOV) = 24 cm, TR/TE1/ΔTE = 48.0/6.3/4.1 ms, number of TEs = 10, FA = 15°, R = 2, voxel size = 0.75 × 0.93 × 3 mm^3^.

Twenty‐two BA patients were scanned on a 3T GE scanner with similar overall imaging protocol, including the axial 3D multi‐echo GRE sequence for QSM. The harmonized QSM imaging protocol has been demonstrated to have high reproducibility across different scanner vendors.^21^ QSM was reconstructed from complex GRE images using a fully automated Morphology Enabled Dipole Inversion algorithm zero‐referenced to the ventricular cerebrospinal fluid (MEDI+0).^22^

### Lesion segmentation and PRL lesion detection

**Figure 2:**
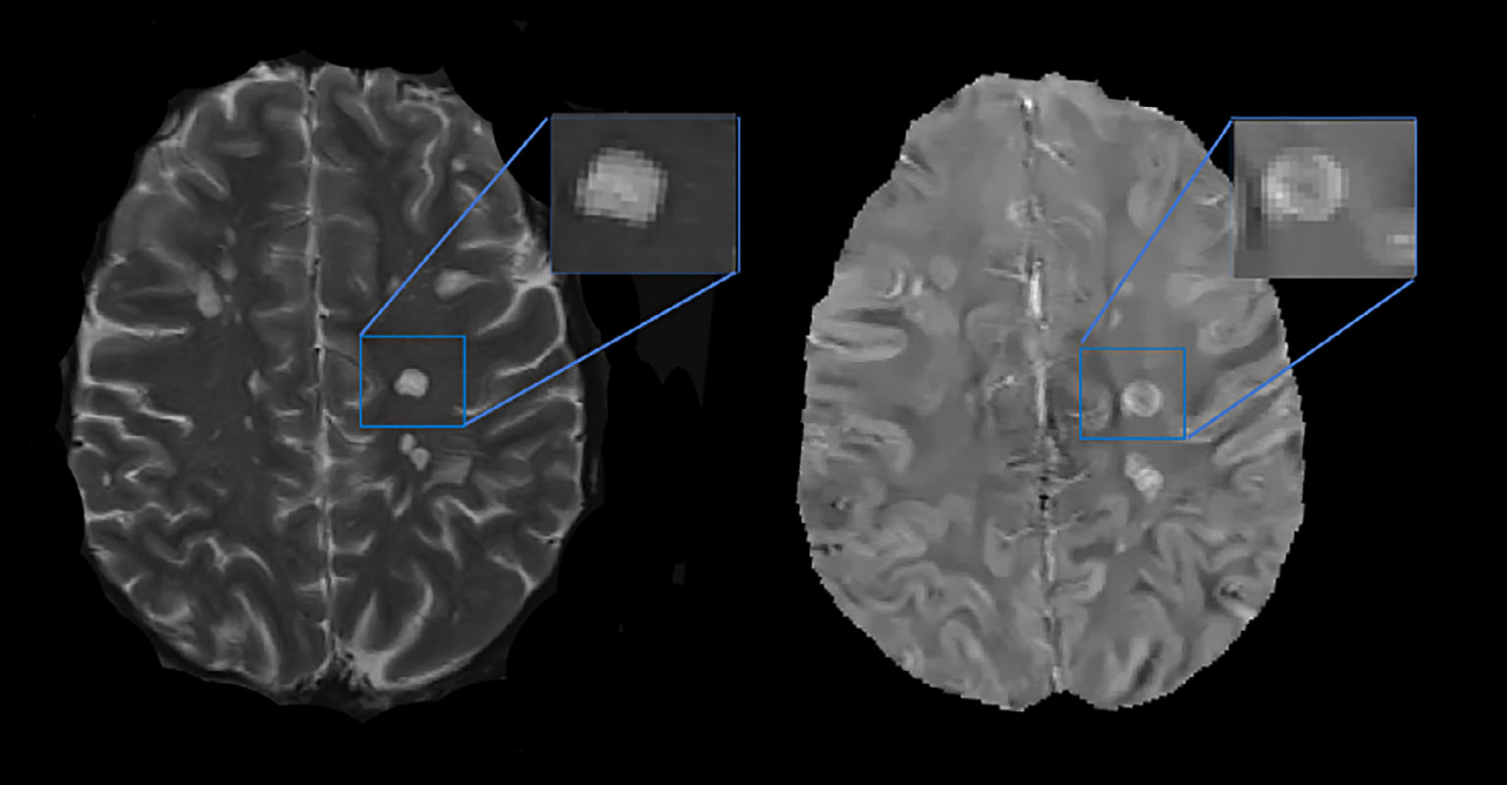
Demonstration of a paramagnetic rim lesion (PRL) on T2w image (left) and QSM (right). There is a clear hyperintense rim on QSM at the periphery of the lesion with hypointense central core. PRL, paramagnetic rim lesion; T2w, T2‐weighted imaging; QSM, quantitative susceptibility mapping.

MS lesions were automatically identified and segmented on the FLAIR image,^23^ which was followed by manual editing and creation of individual lesion labels. T1 and T2 images were referenced, as needed, during the editing process to define lesion boundaries, especially in the case of confluent lesions. The consensus of two blinded reviewers was used to identify PRLs on QSM, and a third independent reviewer resolved any discrepant lesions.^15^ Figure 2 shows a representative example of a PRL with hyperintense appearance on the FLAIR image and a hyperintense rim relative to the lesion core on the QSM image. Lesions with partial or complete rims were considered PRLs, in accordance with the most recent consensus statement on imaging chronic active lesions.^24^ Enhancing lesions were excluded.

### Statistical analysis

**Figure 3:**
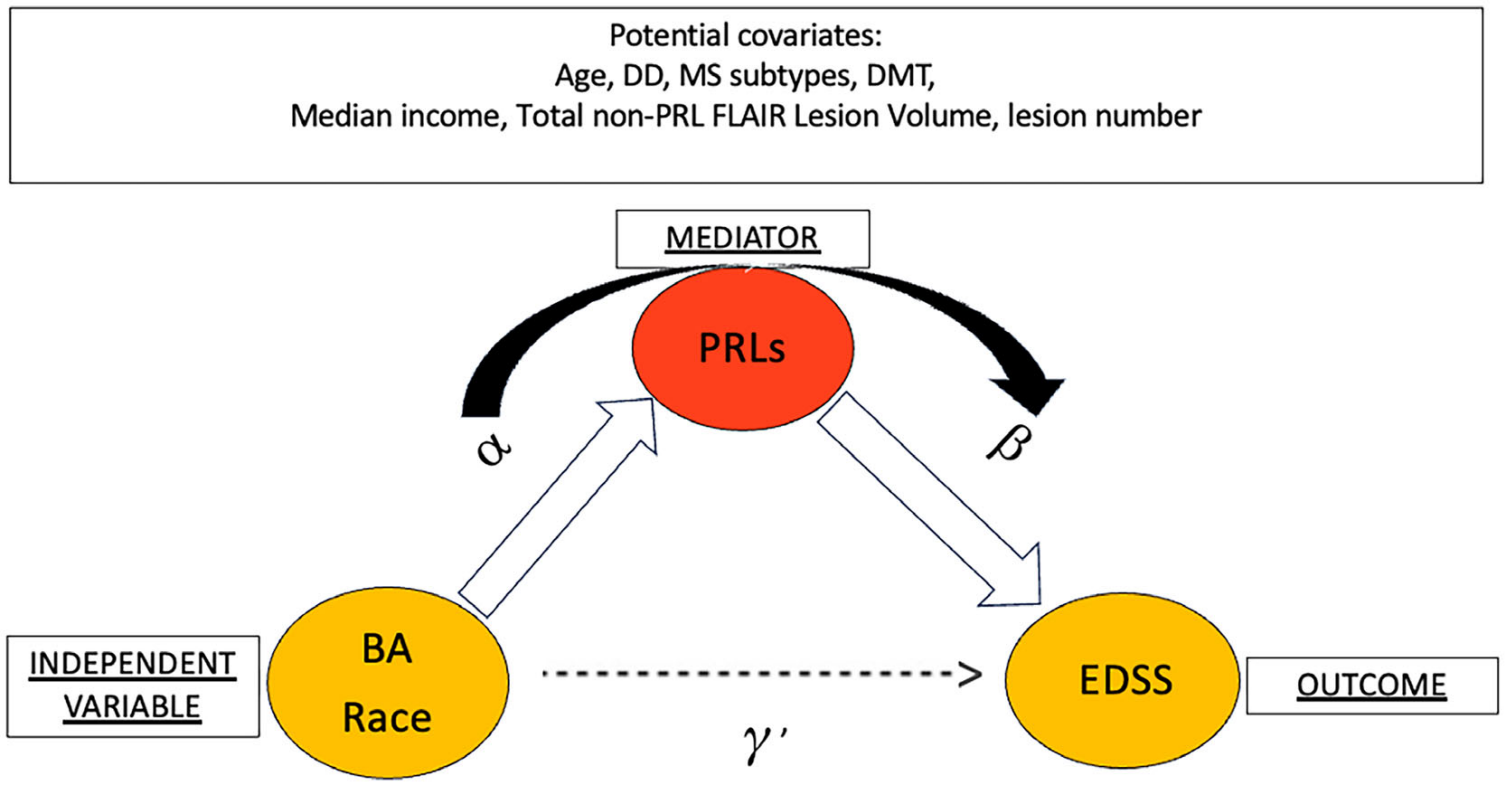
Proposed causal mediation analysis path to assess the impact of PRLs on the observed racial disparities in disability based on EDSS. Path diagram for mediation including EDSS (outcome), race (independent variable), PRLs (mediator), and all possible covariates. Total effect is represented by $\gamma^{\prime }$. α and β are the key parameters of the mediator and outcome models. BA, Black American; PRLs, paramagnetic rim lesions; DMT, disease‐modifying therapy; EDSS, expanded disability status scale; DD, disease duration; MS, multiple sclerosis.

We used a chi‐squared test (with continuity correction) when testing for categorical variables, a two‐sample t‐test (unequal or equal variances) for continuous normal variables, and Kruskal or Wilcoxon tests for nonnormal continuous variables. A Fisher test was used for contingency tables. A model‐based mediation analysis was performed to examine if the percentage of PRLs per total FLAIR lesions mediated the relationship between race and EDSS (Fig. 3). As a prerequisite for mediation analysis, we fit total effect, outcome, and mediator models while adjusting for all observed covariates. The first linear model assessed the relationship between EDSS and race adjusting for patient level covariates including age, sex, disease duration (years), DMT category, non‐PRL FLAIR lesion volume, and median income of patients' zip code. This model tests the total effect of race on EDSS. The second linear model assessed the relationship between EDSS and race while adjusting for the effect of PRLs and all other covariates (outcome model). A final third model was used to test the relationship between PRLs and race while controlling for all other covariates (mediator model).

To test our main hypothesis, we formulated a mediation model representing the path from race to PRLs to EDSS, and estimated the average causal mediation effect (ACME) and average direct effects (ADE) under the assumption of sequential ignorability.^25^, ^26^ Nonparametric bootstrap confidence intervals (25,000 bootstrap samples) with the percentile method were used. We conducted a sensitivity analysis for the sequential ignorability assumption. Sensitivity analysis was conducted by varying the value of the correlation between the residuals of the mediator and outcome and examining their impact on the estimated ACME. A comprehensive description of the causal mediation model is included in Section A of the Supplementary Material. An analysis regarding PRL mediator variable selection is included in Section B of the Supplementary Material. The Supplementary Material file also includes estimation procedure and model output for the final model (Sections C and D). All analysis was performed using R: A language and environment for statistical computing.^27^

## Results

### Patient clinical characteristics

There were 117 BA and 123 WA patients with MS identified for this study (Table 1). There were no statistically significant differences in the two patient cohorts regarding the following clinical covariates: age, sex, disease duration, time from symptom onset to diagnosis, time from symptom onset to treatment start, and DMT allocation (none, lower vs. higher efficacy). At time of MRI scan, 5 BA patients (4%) and 6 WA patients (5%) were reported to have disease activity, based on reported relapse activity (*P* = 0.825). BA patients had a higher level of disability (mean EDSS [SD] = 2.34 [2.11]) as compared to WA patients (mean EDSS [SD] = 1.3 [1.62]). On average, BA patients had a lower mean zip code income as compared to WA patients (*P* < 0.001).

### Race‐related differences in PRLs

Although the number of FLAIR lesions was similar between the two patient cohorts (*P* = 0.487), the total volume of FLAIR lesions was notably higher for BA compared to WA patients, *P* < 0.001. The prevalence of PRLs (i.e., having at least one PRL) was higher in the BA patients (55%) compared to WA patients (39%), *P* = 0.022. In addition, a higher percentage of PRLs among total white matter lesions was observed among BA patients (Table 1). In a linear model (see Table S4), BA patients exhibited an average of 4.65 point increase in the percentage of PRLs (*P* = 0.003) after adjusting for significant covariates (age and DMT).

In a separate linear model (see Table S5), after controlling for significant covariates including age ($\hat{\beta }$: 0.05, *P* < 0.001), DMT: lower versus higher efficacy ($\hat{\beta }$: −0.58, *P* = 0.028), untreated versus higher efficacy ($\hat{\beta }$: −0.31, *P* = 0.344), non‐PRL lesion volume ($\hat{\beta }$: 0.00, *P* = 0.55), and percentage of PRLs ($\hat{\beta }$: 0.02, *P* = 0.031), BA patients had, on average, a 1.04‐point higher EDSS (*P* < 0.001) compared to WA patients.

### PRLs as a mediator in race‐related differences in EDSS

**Figure 4:**
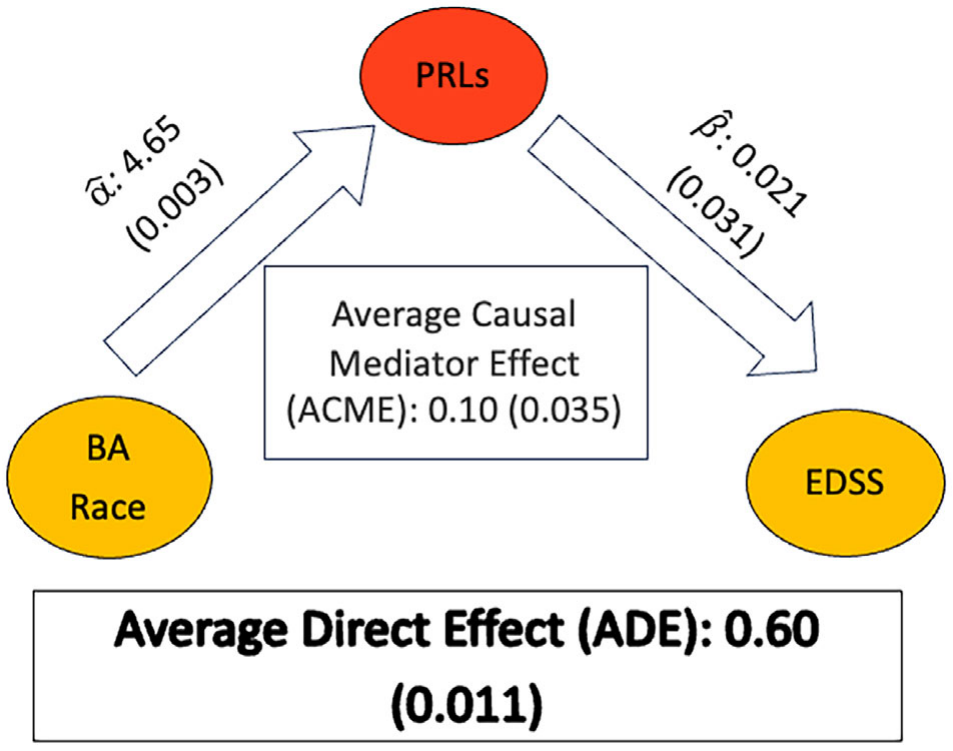
Estimated parameters from the mediation analysis. Estimates of the size of the effects (*P*‐values) are provided. Marginal effects are estimated from a linear model with age, DMT (lower efficacy vs. none), and non‐PRL lesion volume as additional covariates (all *P*‐values <0.05). $\hat{\alpha }$: =4.65 (*P* = 0.0034) and $\hat{\beta }$: =0.021 (*P* = 0.031). BA, Black American; PRLs, paramagnetic rim lesions; DMT, disease‐modifying therapy; EDSS, expanded disability status scale; ACME, average causal mediation effect; ADE, average direct effect.

Figure 4 shows the parameter estimates, mediation effect, and direct effect for the parsimonious final model assessing the impact of PRLs on the observed racial disparities in EDSS. In this model, the percentage of PRLs was identified as a significant mediator (*P* = 0.031) in the relationship between race and EDSS, with an ACME of 0.10 (95% bootstrap confidence interval: [0.0049–0.24]) while controlling for the following outcome model covariates: age (*P* < 0.001), DMT (lower/med *P* = 0.028; untreated *P* = 0.344), and non‐PRL lesion volume (*P* < 0.001). Furthermore, there was a significant direct effect of race on EDSS, denoted as the ADE of 0.60 (95% bootstrap confidence interval: [0.136–1.05], *P* = 0.0114), and an overall significant total effect (*P* = 0.003).

In summary, the estimated proportion of PRL mediation effect on the relationship between BA race and EDSS is 0.141, *P* = 0.0379 (95% bootstrap = 0.0063–0.490), that is, the proportion of PRLs accounted for 14.09% of the total effect of race on disability (*P* = 0.0379).

We conducted a sensitivity analysis for the possibility of unobserved confounders with respect to the percentage of PRLs. We used the correlation, ρ, between the residuals of the mediator and outcome models. The sensitivity analysis showed a narrow sensitivity region for ρ between 0.1 and 0.2. That is, the ACME would be zero if the unobserved confounders affecting EDSS and percentage of PRLs account for correlations between the residuals of the mediator and outcome models in the 0.1 and 0.2 range.

## Discussion

Greater disability and faster disease progression are known to occur in BAs compared to WAs with MS.^4^ Recent advances in MRI have also shown that PRLs can be used as imaging biomarkers to indicate levels of chronic active inflammation, which may be used as prognostic factors for disease course.^28^ However, there has been a lack of research in specifically associating the presence of PRLs with disability in BAs, which generally is a historically understudied population. Here, for the first time, we try to bridge this gap using QSM to identify lesions that retain a rim of iron‐laden microglia/macrophages and represent a subset of CALs.

The primary objective of our study was to assess the role of PRLs as a possible mediator for higher disability in the BA MS population, as opposed to demonstrating mere association or attempting to construct a comprehensive predictive model with all possible variables. First, we validated previous research that indicated there was increased disability among Black American (BA) patients with MS. Consistent with this, we demonstrated larger lesion volumes in these patients, supporting a more severe phenotype within this racial subgroup. To assess the influence of non‐PRLs, we excluded the volume of PRLs from the standard total lesion volume variable (see Supplementary Material for statistical analysis) and observed an association between non‐PRL burden and higher disability in BA individuals in the linear models. All significant covariates were included in the models, however, assessing the potential for all other covariates as mediators was beyond the scope of this work. Importantly, PRLs are likely one of several mediators influencing the relationship between race and disability. We believe that this key observation could greatly advance the field's understanding of MS disparities and imaging biomarkers.

In vivo studies with GRE MRI have demonstrated that PRLs can be detected in the preclinical stage of the disease, such as in radiologically isolated syndrome (RIS),^29^ pediatric MS,^30^ and broadly throughout the relapsing phase of the disease.^31^ Across multiple studies, in the broader MS patient population, about 35–60% of patients with MS have at least one PRL.^31^ The factors influencing the development of these lesions in specific individuals, however, remain unclear. Several studies^14^, ^16^, ^32^, ^33^ including ours^15^, ^34^ have confirmed the negative impact of PRLs on disability and have been recognized as a marker of a more aggressive disease. Given the diverse pathological mechanisms contributing to MS progression, which likely vary among individuals, it is essential to consider each patient's disease trajectory in the context of the predominant pathological mechanisms.^2^ Taking these findings into account, both a patient's race and the percentage of PRLs should be considered when formulating treatment strategies. More specifically, there is a good motivation for adopting a more proactive approach to treatment, particularly with higher efficacy DMTs, aimed at averting the occurrence of PRLs and the subsequent onset of disability, which has been noted to occur at a younger age in BA patients.^35^ Indeed, in BAs that were treated with lower efficacy DMTs such as interferon‐beta‐1 (EVIDENCE trial), there were more frequent MS exacerbations and a greater number of new MS lesions at 48 weeks compared to WA patients.^36^ While anti‐CD20 depleting treatments did not demonstrate a therapeutic effect on PRLs,^17^ our work has revealed a reduction of innate immune activity on QSM in PRLs in patients treated with dimethyl fumarate.^37^ This suggests the potential for existing DMTs to influence innate immune activity. Moreover, as future therapies are being developed to target CNS compartmentalized inflammation, these results may offer an opportunity for new approaches to MS treatment algorithms.

We sought to analyze the role of socioeconomic factors in order to account for other reasons underlying the racial disparity with disability in MS. Minority populations, such as with BAs, may have more difficulty with accessing specialists or face additional barriers such as lower income, limited insurance, less education, and less computer literacy.^38^ In order to approximate these socioeconomic barriers, zip codes with associated income levels were incorporated in the statistical analysis. Of note, income level was not a significant covariate. We were not able to collect comprehensive educational‐level data, which is a limitation of this analysis. Race was determined by self‐report, and we were not able to perform genetic ancestry testing to determine ethnic variations. Other limitations of the study were its retrospective and cross‐sectional design, which predisposes it to bias and perhaps unrecognized confounders. While there were no statistically significant differences in demographics, clinical characteristics and treatment regimens between the characteristics of our two cohorts, except for higher EDSS scores in BA cohort, the patient data were collected from a single center. In addition, both cohorts were predominantly RRMS patients with a relatively benign disease course, having a low lesion number, low lesion volume, and low EDSS. The percentage of lesions with PRLs was also generally low, particularly in the WA cohort. These factors should be considered, as they may limit the generalizability of our results and may not extend to more disabled MS patients. Importantly, there are missing variables known to be associated with disability, such as measures of volume changes in the brain and spinal cord disease. These could potentially be significant covariates in the association between EDSS and race, as part of the mediation process, and their absence is a limitation of our work. Unfortunately, due to the lack of available spinal cord data and the inconsistency in brain imaging platforms, we were unable to incorporate this into our analysis. Future work with more comprehensive MRI features for all patients would be ideal. Moreover, confirmation of our findings would necessitate additional studies involving larger cohorts and patients from multiple centers. Furthermore, future longitudinal studies will offer additional data to elucidate the relationship between the tendency of BA patients to develop PRLs and the impact of these lesions on disability progression.

In conclusion, our study establishes a causal relationship between PRLs and race‐related disability disparities among BA and WA patients with MS. These findings enhance our understanding of race‐related differences in disease severity. The exact mechanisms underlying this greater disease activity in BA cohorts is unclear but, as we learn more about the pathological drivers of PRLs, this may provide new insights into the disease overall. This should also guide future studies aimed at preventing the higher disability and faster disease progression observed within the often‐understudied BA MS patient population.

## Author Contributions

NMM, SAG, SHR, and UWK contributed to the conception and design of the study. MM, IP‐B, and KM contributed to acquisition of the data. TDN contributed to analysis of the data. NMM and SAG drafted the manuscript and preSpared the figures. All authors were involved in editing for intellectual content and readability, and in approval of the final version. SAG takes full responsibility for the data, analysis, and interpretation reported in this study and for the conduct of the research. SAG has full access to the data and the right to publish any and all data, separate and apart from the guidance of any sponsor.

## Conflict of Interest

All authors have submitted their disclosure forms as part of submission. Under a program agreement between MGH and Biogen, Dr. Michaelson currently spends approximately half of her time working at Biogen training in the area of therapeutics development. Biogen pays a portion of her salary to MGH for the time she works there. Dr. Michaelson's interests were reviewed and are managed by MGH and Mass General Brigham in accordance with their conflict of interest policies. Her work at Biogen is unrelated to this current manuscript, which was conducted prior to starting at Biogen. Dr. Hurtado Rúa receives grant funding from NIH/NIDS and the National Multiple Sclerosis Society. Dr. Kaunzner has no conflicts to disclose. Dr. Nguyen has no conflicts to disclose. Dr. Gauthier receives grant support from the National Multiple Sclerosis Society, NIH, NINDS, Genentech and receives consulting fees from Biogen and Contineum Therapeutics. Sponsors did not have a role in the study design, in the collection, analysis, interpretation of data, in the writing of the report, or in the decision to submit the article for publication.

## Supporting information


**Figure S1.** Proposed causal mediation analysis path to assess the impact of PRLs on the observed racial disparities in disability based on EDSS.
**Table S1.** Comparison of mediator variable candidates using correlational evidence and an initial path analysis.
**Table S2.** Description of modeling steps.
**Table S3.** Parameter estimates – total effect model for EDSS.
**Table S4.** Parameter estimates – mediator model.
**Table S5.** Parameter estimates – outcome model.
**Figure S2.** Estimated direct and indirect effects with their respective 95% bootstrap confidence intervals and *P*‐values.

## Data Availability

Anonymized data not published within this article will be made available by request from any qualified investigator.
