# Correction to Impact of paramagnetic rim lesions on disability and race in multiple sclerosis: mediation analysis

**DOI:** 10.1002/acn3.52223

**Published:** 2024-10-07

Michaelson NM, Hurtado Rúa S, Kaunzner UW, Marcille M, Pliska‐Bloch I, Markowitz K, Nguyen TD, Gauthier SA. Impact of paramagnetic rim lesions on disability and race in multiple sclerosis: mediation analysis. Ann Clin Transl Neurol. 2024 Sep 17. doi: 10.1002/acn3.52203. Epub ahead of print. PMID: 39290047.

One of the authors was incorrectly listed as ‘Sandra H. Rua’ but it should instead be Sandra Hurtado Rúa.

We apologize for this error.

