# Supplementary material for: Impact of paramagnetic rim lesions on disability and race in multiple sclerosis: mediation analysis
Source: Ann Clin Transl Neurol. 2024 Sep 17;11(11):2923–31. doi: 10.1002/acn3.52203 (PMC11572731; doi:10.1002/acn3.52203)
Supplement: Supplementary file 1 — Figure S1. Proposed causal mediation analysis path to assess the impact of PRLs on the observed racial disparities in disability based on EDSS. Table S1. Comparison of mediator variable candidates using correlational evidence and an initial path analysis. Table S2. Description of modeling steps. Table S3. Parameter estimates – total effect model for EDSS. Table S4. Parameter estimates – mediator model. Table S5. Parameter estimates – outcome model. Figure S2. Estimated direct and indirect effects with their respective 95% bootstrap confidence intervals and P‐values. [file ACN3-11-2923-s001.docx]

**Title: Impact of Paramagnetic Rim Lesions on Disability and Race in Multiple Sclerosis: Mediation Analysis**

**Supplementary Material**

**Running Title: Impact of Paramagnetic Rim Lesions on Disability**

Nara Miriam Michaelson, MD, MS^1^, Sandra Hurtado Rúa, PhD^2^, Ulrike W. Kaunzner, MD, PhD^1^, Melanie Marcille, BS^1^, Iliana Pliska-Bloch, BA^1^, Kimberly Markowitz, BA^1^, Thanh D. Nguyen, PhD^3^, and Susan A. Gauthier, DO, MPH^1,3,4^

^1^Department of Neurology, Weill Cornell Medicine, New York, NY, USA

^2^Department of Mathematics and Statistics, Cleveland State University, Cleveland, OH, USA

^3^Department of Radiology, Weill Cornell Medicine, New York, NY, USA

^4^Feil Family Brain and Mind Institute, Weill Cornell Medicine, New York, NY USA

Corresponding Author:

Susan Gauthier, DO, MPH

Judith Jaffe Multiple Sclerosis Center

1305 York Avenue

New York 10021, NY

Tel: (646) 962-9800

Fax: (646) 962-0390

Email: [sag2015@med.cornell.edu](mailto:sag2015@med.cornell.edu)

Number of characters in the title: 99

Number of characters in running head: 48

Number of words in the abstract: 250

Number of words in body of the manuscript: 3,252

Number of figures and tables: 5

1. **Mediation Model**

Causal mediation analysis is a statistical method used to explore the mechanisms through which an independent variable (Race) influences a dependent variable (EDSS). This type of analysis involves breaking down the total effect of the independent variable on the dependent variable into direct and indirect effects through a mediator variable (percentage of PRLs). We follow a model-based inference approach, the standard practice in the mediation analysis to date.^36^



Selecting a mediator variable in a mediation analysis involves careful theoretical and empirical considerations. We used percentage of PRLs as the mediator variable (see Section B of this document for rationale about mediator selection) representing the contribution of paramagnetic rim lesions to the relationship between race and disability. Specifically, after adjusting for other covariates, the hypotheses of interest are:

H1: Race influences the percentage of PRL (as measure of the contribution of paramagnetic rim lesions)

H2: The percentage of PRLs influences disability as measured by EDSS

H3: Race influences disability (EDSS) through the percentage of PRLs


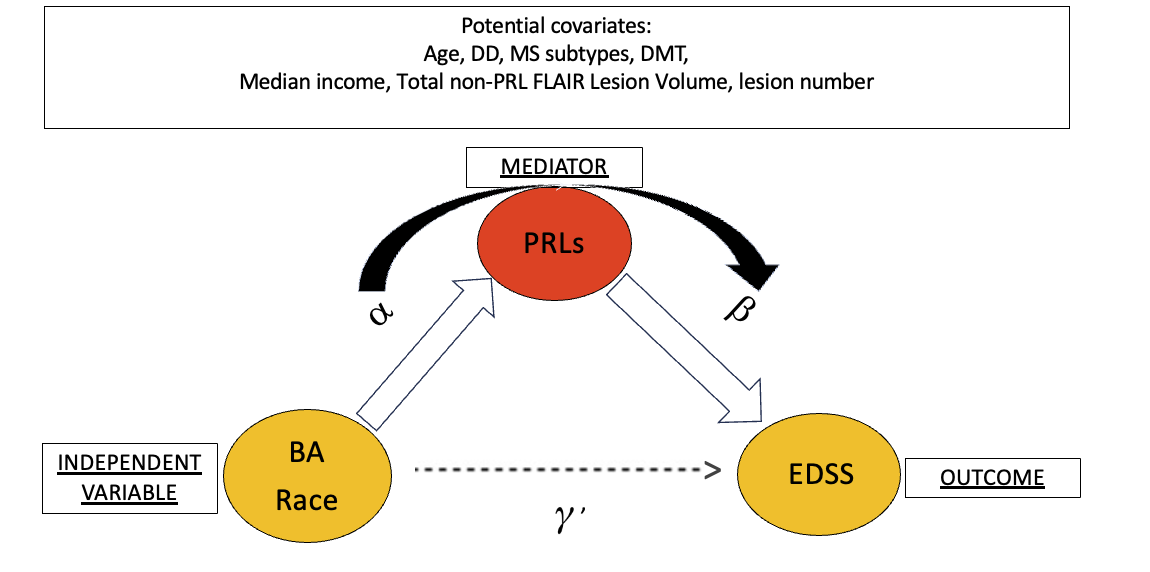
 **Figure S1**. Proposed Causal Mediation Analysis Path to Assess the Impact of PRLs on the Observed Racial Disparities in Disability Based on EDSS. Path diagram for Mediation including EDSS (Outcome), Race (Independent variable), PRLs (Mediator) and all possible covariates. Total effect is represented by
$\gamma^{'},$ while $\alpha, \beta,$are the key parameters of the mediator and outcome models, respectively. Abbreviations: BA, Black American; PRLs, paramagnetic rim lesions; DMT, disease modifying therapy; EDSS, expanded disability status scale.

Model represented by figure S1 and parametrized by equations (1) to (3) is a traditional mediation model. Equation 1 represents the total effect model (race to EDSS) while Equations 2 and 3 represent the mediator and outcome models, respectively. Equation 2 models the direct path from race to PRLs accounting for all other covariates, while equation 3 models the association between race and EDSS while adjusting for percentage of PRLs as well as other covariates.

$EDSS= {\gamma'}_{0}+ {\gamma'}_{1} X_{1}+{\gamma'}_{2} X_{2} +\ldots+ {\gamma'}_{8} X_{8}+\gamma' Race+\varepsilon$ , (1)

$PRL\%= \alpha_{0}+ \alpha_{1} X_{1}+\alpha_{2} X_{2} +\ldots+\alpha_{8} X_{8}+ \alpha Race+ \varepsilon_{PRL}$ , (2)

$EDSS= \gamma_{0}+ \gamma_{1} X_{1}+\gamma_{2} X_{2} +\ldots+ \gamma_{8} X_{8}+\gamma Race+ \beta PRL\%+\varepsilon_{EDSS},$ (3)

Where $X_{1}\ldots. X_{8}$represent all observed clinical, socioeconomical and MRI patient level covariates, including sex, DD, age, MS subtypes, DMT, median income, time to diagnosis, and time to treatment. The parameter $\gamma'$ represents the total effect of Race on EDSS.

Equation 4 represents the mediation path from race to PRLs to EDSS. This mediation model assumes causal direction (additive effects and sequential ignorability assumption), and standard regression assumptions (linearity, independence, normality, and no collinearity). We check standard regression assumptions and perform a sensitivity analysis to the sequential ignorability assumption.

$EDSS = {\gamma''}_{0}+ {\gamma''}_{1} X_{1}+{\gamma''}_{2} X_{2} +\ldots+ {\gamma''}_{8} X_{8}+\left( \gamma+\alpha\beta\right)Race+ \alpha\beta PRL\%+ \beta\varepsilon_{PRL} +\varepsilon_{EDSS}$ , (4)

Where $X_{1}\ldots. X_{8}$represent all observed clinical, socioeconomical and MRI patient level covariates, the parameter γ represents the direct effect of Race on EDSS and αβ represents the indirect effect. Note that some variables were transformed to improve normality and linearity when needed.

1. **Possible PRL Related Mediators:**

Selecting a mediator variable in a mediation analysis involves careful theoretical and empirical considerations that answer a specific research question. The goal of this research is to study if paramagnetic rim lesions contribute to the relationship between race and disability. In this context, several other possible MRI derived variables (percentage of PRLs, number of PRLs, presence of PRLs, or lesion volume of PRLs) could have been used to mediate the relationship between disability and race.

We used two statistical criteria to select the best mediator variable candidates: Correlational evidence and an initial path analysis. Correlational evidence was assessed by computing the Kendall coefficient and point-biserial correlation. The initial path analysis tests the univariate linear relationships without covariate adjustment. Based upon the results of Table S1, PRL’s candidates to mediate the relationship between Race and EDSS are percentage of PRL and Presence of at least one PRL (All p-values < 0.05). The percentage of PRLs was selected as the mediator given that the “Presence of at least one PRL” (a binary variable) would require a generalized linear model, a technique that has more assumptions, is more computational demanding, less robust to outliers, and less simple to interpret.

| **Possible PRL-related Mediator Variables** | **Correlation Analysis** | | **Initial Path Analysis** | |
| --- | --- | --- | --- | --- |
|  | EDSS:  τ (p-val) | Race:  $r_{pb}$ (p-val) | Race → Mediator:  (p-val) | Mediator → EDSS:  (p-val) |
| Percentage of PRLs | 0.1215  (**0.0198**) | 0.1873 (**0.0037**) | (**0.0037**) | (**0.0307**) |
| Number of PRLs | 0.1365 (0.0108) | 0.0947 (0.1450) | (0.1450) | (0.3410) |
| Presence of at least one PRL (Y/N) | 0.1625 (**0.0047**) | 0.1511 (**0.0190**) | (**0.0203**) | (**0.0245**) |
| FLAIR PRL volume (PRL) | 0.0577 (0.2804) | 0.0457 (0.4821) | 0.0457  (0.4820) | (0.0165) |

**Table S1.** Comparison of mediator variable candidates using correlational evidence and an initial path analysis. The Kendall rank correlation tau statistic (τ) is used to estimate a rank-based measure of association. Biserial correlations are used to estimate correlation between a categorical and a quantitative variable. Two-sided p-values are reported.

Importantly, a mediator variable needs to be distinct from any covariate to avoid multicollinearity issues in the outcome model. It also needs to be defined in consistency with the sequential ignorability assumption. As a result, covariates should be only included in the model to adjust for confounding effects. Throughout the analysis we also check for model robustness, that is, linearity, normality, and collinearity. For example, total FLAIR Lesion Volume and total non-PRL FLAIR Lesion Volume have large correlation (r=0.970), however, the latter is not correlated with percentage of PRLs (r=-0.003). As a result, in accordance with the model assumptions, total non-PRL FLAIR Lesion Volume would be preferred to use as a covariate in a model that assumes percentage of PRLs as mediators.

1. **Estimation Procedure**

| **Table S2.** | **Description of Modeling Steps** |
| --- | --- |
| **Step 1:**  **Formulate Initial Models** | Mediation analysis requires a statistically significant pathway model (See Figure S1). The aim of Step 1 is to confirm that race is statistically significant for each pathway as well as having a statistically significant covariates for the total effect model. We use linear regression models and find the best models (Figure S1, Eq. 1 to 3). We used stepwise regression with backward elimination to select the most important predictors for each model. Final models include all statistically significant predictors.   - As a prerequisite of mediation analysis, we fit the total effect model (Eq. 1) to test the association between EDSS and race while controlling for sex, DD, age, MS subtypes, DMT, Total non-PRL FLAIR Lesion Volume, total lesion number, and median income.   - Initial check for multicollinearity.   - Initial check of the residual plots (linearity, heteroscedasticity, and normality)   - Report final model (statistically significant covariates).   - Check statistically significant variables and check that Race is a significant variable. - Fit the mediator model (Eq. 2) using all observed covariates.   - Initial check for multicollinearity.   - Initial check of the residual plots (linearity, heteroscedasticity, and normality)   - Check significant variables. At the 5% significant level, the mediator model includes the following statistically significant variables: Race, Age, DMT, and Total non-PRL FLAIR Lesion Volume - Fit the outcome model (Eq. 3) using all observed covariates.   - Initial check for multicollinearity.   - Initial check of the residual plots (linearity, heteroscedasticity, and normality)   - Check significant variables. |
| **Step 2:**  **Estimate model parameters (Eq. 4)** | Mediation analysis was used to investigate whether PRL acts as mediator in the relationship between Race and EDSS.   - Use White’s heteroskedasticity-consistent estimator for the covariance matrix if heteroscedasticity of the errors is suspected. - We use nonparametric Bootstrap Confidence Intervals with the Percentile Method (25000 simulations) to estimate the parameters and their confidence (Confidence intervals and significant). - We evaluate the significance and size of the indirect effect to determine whether mediation is present. |
| **Step 3:**  **Estimate ACME, ADE** | - The direct and indirect effects are reported with their respective confidence intervals and p-values.   - Estimate the Average Causal Mediation Effect (ACME)   - Estimate the Direct Effect (ADE)   - Declare whether the percentage of PRLs is a mediator. - Final models and plots are reported (Figure S2). |
| **Step 4:**  **Model robustness** | - Linearity was checked using scatter plots. - Independence was assumed. - Normality was checked by plotting normality plots. - Collinearity was assessed by using generalized variance inflation factors. |
| **Step 5:**  **Sensitivity Analysis** | Conduct sensitivity analyses to assess the robustness of ACME to different modeling assumptions.   - The sensitivity parameter, representing the correlation between the residuals of the mediator and outcome regressions, was set to vary from −0.9 to 0.9 by 0.1 increments. - Explore the impact of potential unobserved confounders. |

Our primary objective was to test whether PRLs mediate higher EDSS in the BA population. The mediator and outcome models were fitted using linear models. The average causal mediation effect (ACME) and average direct effects (ADE) were estimated under the sequential ignorability assumption [24, 37] Nonparametric Bootstrap Confidence Intervals (25,000 Bootstrap samples) with the Percentile Method were used.

We conducted a sensitivity analysis for the sequential ignorability assumption. Sensitivity analysis is conducted by varying the value of the correlation between the residuals of the mediator and outcome and examining their impact on the estimated ACME. All analysis were performed using R: A language and environment for statistical computing.24 Table S3 list the steps we considered to estimate model parameters.

1. **PRLS % as mediator- Model Results**

**Table S3.** Parameter estimates - Total Effect Model for EDSS

**
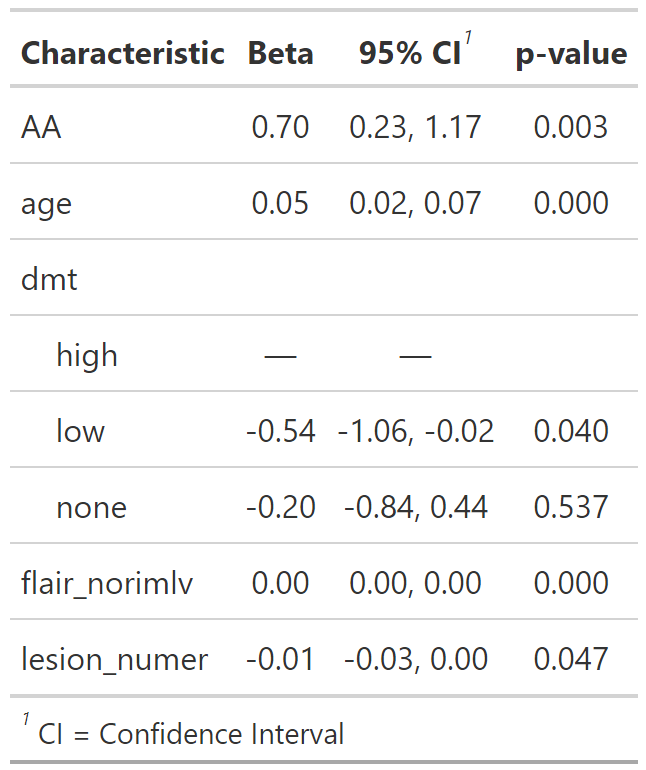
**

**Table S4.** Parameter estimates - Mediator Model

**
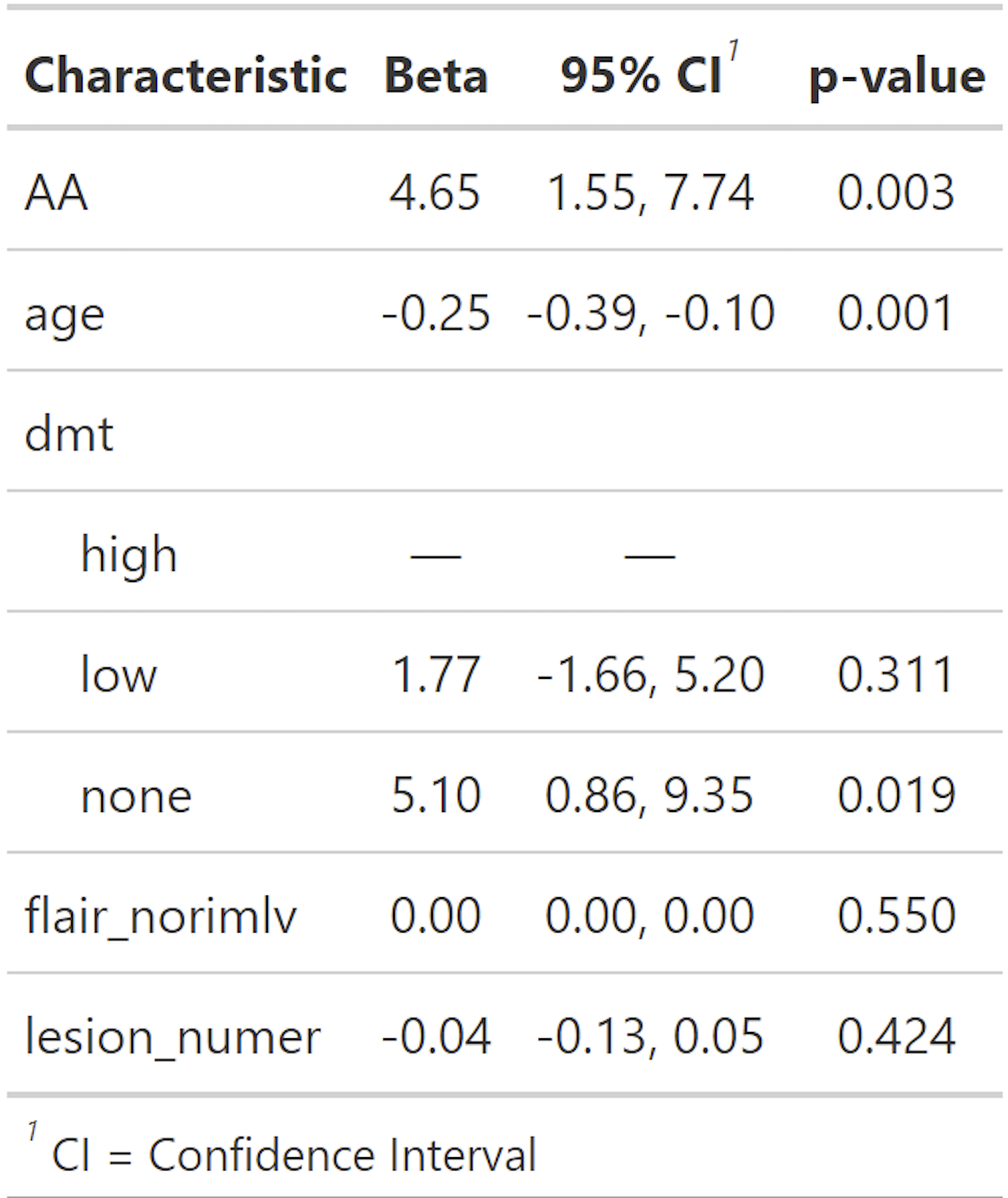
**

**Table S5.** Parameter estimates - Outcome Model


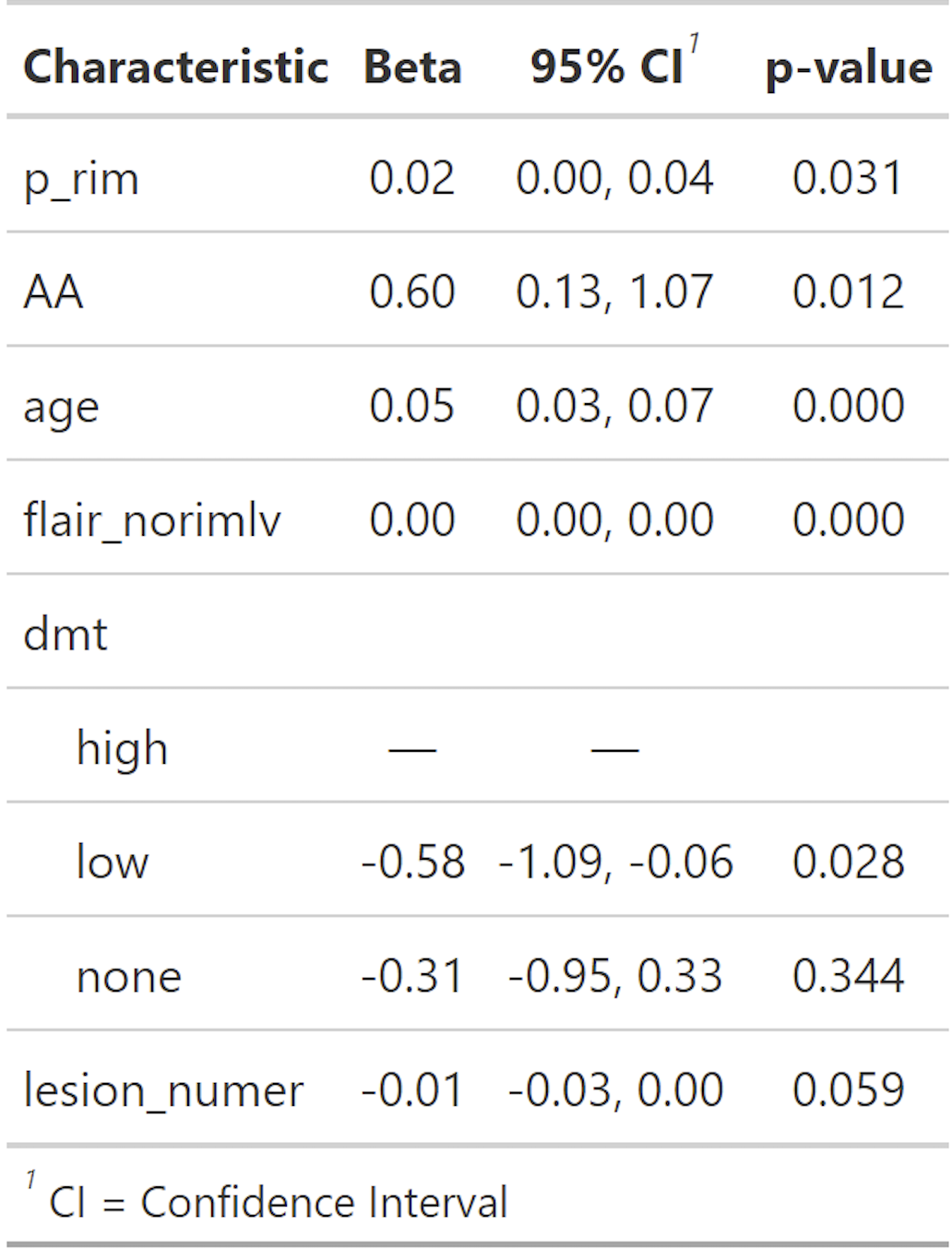


**Figure S2.** Estimated direct and indirect effects with their respective 95% Bootstrap confidence intervals and p-values.


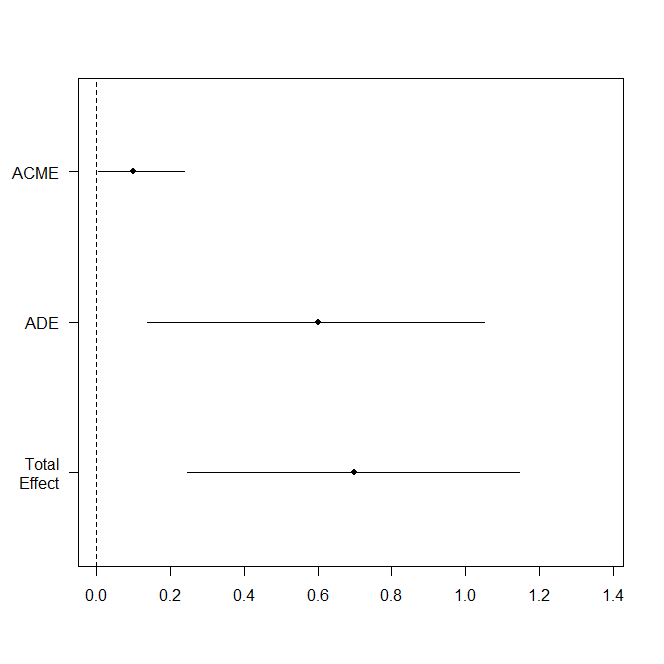


References

24. Tingley D, Yamamoto T, Hirose K, Keele L, Imai K. mediation: R Package for Causal Mediation Analysis. J Stat Soft. 2014;59:1–38.

25. R Core Team. R: A Language and Environment for Statistical Computing [online]. Vienna, Austria; 2023. Accessed at: https://www.R-project.org/.

37. Kosuke Imai, Luke Keele and Teppei Yamamoto (2010). Identification, Inference and Sensitivity Analysis for Causal Mediation Effects. Statistical Science, 25(1), 51-71.
